# Using a chimeric respiratory chain and EPR spectroscopy to determine the origin of semiquinone species previously assigned to mitochondrial complex I

**DOI:** 10.1186/s12915-020-00768-6

**Published:** 2020-05-20

**Authors:** John J. Wright, Justin G. Fedor, Judy Hirst, Maxie M. Roessler

**Affiliations:** 1 School of Biological and Chemical Sciences, Queen Mary University of London, Mile End Road, London, E1 4NS UK; 2 Medical Research Council Mitochondrial Biology Unit, University of Cambridge, Cambridge, CB2 0XY UK; 3 Department of Chemistry, Imperial College London, Molecular Sciences Research Hub, White City Campus, Wood Lane, London, W12 0BZ UK

**Keywords:** NADH:ubiquinone oxidoreductase, Respiratory complex I, Semiquinones, Electron paramagnetic resonance

## Abstract

**Background:**

For decades, semiquinone intermediates have been suggested to play an essential role in catalysis by one of the most enigmatic proton-pumping enzymes, respiratory complex I, and different mechanisms have been proposed on their basis. However, the difficulty in investigating complex I semiquinones, due to the many different enzymes embedded in the inner mitochondrial membrane, has resulted in an ambiguous picture and no consensus.

**Results:**

In this paper, we re-examine the highly debated origin of semiquinone species in mitochondrial membranes using a novel approach. Our combination of a semi-artificial chimeric respiratory chain with pulse EPR spectroscopy (HYSCORE) has enabled us to conclude, unambiguously and for the first time, that the majority of the semiquinones observed in mitochondrial membranes originate from complex III. We also identify a minor contribution from complex II.

**Conclusions:**

We are unable to attribute any semiquinone signals unambiguously to complex I and, reconciling our observations with much of the previous literature, conclude that they are likely to have been misattributed to it. We note that, for this earlier work, the tools we have relied on here to deconvolute overlapping EPR signals were not available. Proposals for the mechanism of complex I based on the EPR signals of semiquinone species observed in mitochondrial membranes should thus be treated with caution until future work has succeeded in isolating any complex I semiquinone EPR spectroscopic signatures present.

**Supplementary information:**

**Supplementary information** accompanies this paper at 10.1186/s12915-020-00768-6.

## Background

Respiratory complex I (NADH:ubiquinone oxidoreductase) is a crucial component of the mitochondrial electron transport chain and thus plays a central role in energy conversion and cellular metabolism [1–3]. Complex I catalyses the oxidation of NADH and the reduction of ubiquinone, coupled to the translocation of four protons across the inner mitochondrial membrane in a mechanism that remains enigmatic, despite recent structural advancements [4]. The current consensus is that the binding and reduction of ubiquinone form an important mechanistic element, but while mechanisms for the long-range coupling that links electron transfer to proton translocation have been proposed on the basis of computational models [5–7], direct experimental evidence for them is lacking.

The key ubiquinone reduction step in catalysis must proceed via a semiquinone (SQ) radical, given that the iron-sulphur (Fe-S) clusters linking the two substrate binding sites are single electron carriers. It is thus no surprise that, over the past four decades, electron paramagnetic resonance (EPR) spectroscopy has been used extensively to investigate the SQ species associated with complex I. However, despite numerous reports of SQ catalytic intermediates forming during turnover [8–12], fundamental disagreements and inconsistencies have led to ambiguity about the importance of SQs in the energy coupling reaction of complex I [13]. Indeed, the stability of the SQs generated during turnover has recently been questioned [14], suggesting that SQs formed do not, in fact, accumulate sufficiently to enable them to be trapped and observed on an experimentally accessible timescale. Given that different mechanisms have been proposed on the basis of different ubiquinone reduction pathways [15–17], a re-investigation of the putative SQs associated with complex I is now warranted.

Most studies of complex I SQs have been conducted in mammalian submitochondrial particles (SMPs), inverted membrane vesicles that both maintain the structural integrity of the inner mitochondrial membrane and allow the NADH substrate to be provided directly for complex I catalysis in vitro. However, SMPs contain the entire complement of inner membrane enzymes (including many enzymes not directly associated with the electron transfer chain) and so any EPR signals observed are difficult to assign to specific enzymes. Assignments of SQs have relied on (1) the inhibition of enzymes downstream of complex I, removing SQs from their sites but invoking secondary effects through inhibition of turnover and alteration of the redox status of the Q-pool, and (2) an exogenous ubiquinone analogue (Q_1_) [10, 11], which has subsequently been shown to also react adventitiously at the complex I flavin (NADH binding) site [18]. Robust investigations of complex I SQs thus require sustained turnover conditions that both replenish the Q-pool and rely on only the natural, highly hydrophobic ubiquinone substrate.

Two SQs are typically attributed to complex I in SMPs, distinguished on the basis of their relaxation properties obtained from EPR measurements: fast-relaxing (SQ_Nf_) and slow-relaxing (SQ_Ns_) [10, 11]. SQ_Nf_ is reported to form only when a substantial proton-motive force (Δp) is present across the inner mitochondrial membrane [10], and to interact with the nearby terminal Fe-S cluster (N2) [8, 19]. However, very particular conditions have been reported to be required for the clear observation of SQ_Nf_ and the SQ-N2 interaction. SQ_Ns_ has been reported in SMPs in the absence of Δp [9] and assigned to complex I based on its sensitivity to complex I Q-site inhibitors, and because it is absent when complex I is in the deactive state [8]. Notably, neither signal has been clearly observed in any purified enzyme system, so that their assignments to complex I have never been proven. Investigations using complex I in proteoliposomes [12, 20] have only provided much lower SQ signal intensities and occupancies than observed in SMPs: < 0.1 μM SQ species and ~ 2% total occupancy [12], compared to ~ 2.5 μM and ~ 100% occupancy [10] in SMPs. The lack of clear evidence for the assignment of observed SQ species to complex I in SMPs demands further investigation into their origins.

**Fig. 1:**
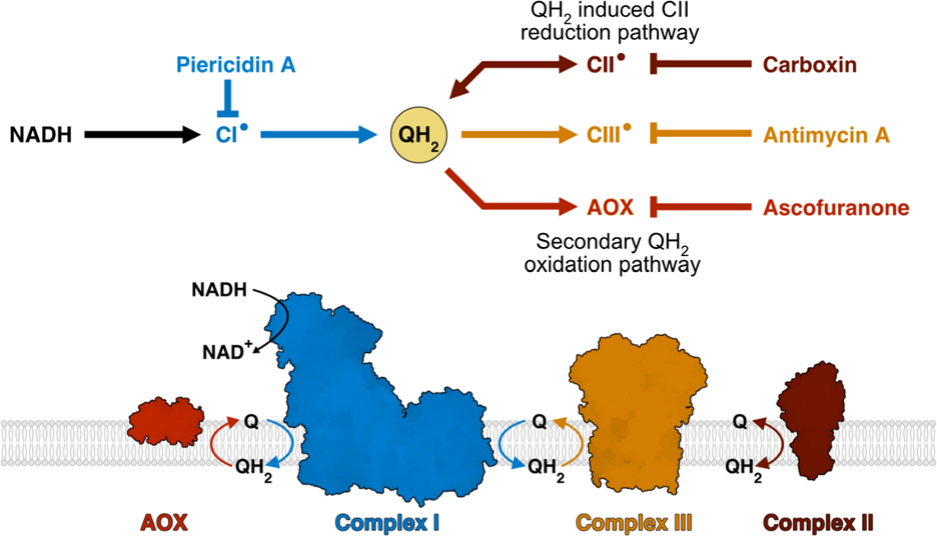
Quinone cycling in AOX-SMPs during NADH oxidation. (Top) Flow diagram showing the routes of quinol (QH_2_) production and oxidation and their inhibitor sensitivities. Radicals (•) indicate expected formation of semiquinones. (Bottom) AOX-SMPs support NADH:O_2_ turnover through both the canonical chain and the complex I-AOX pathway

Here, we used the hitherto-unexploited approach of constructing a chimeric respiratory chain from SMPs and an alternative ubiquinol oxidase, to enable targeted inhibition of enzymes downstream of complex I while maintaining its turnover with the native ubiquinone substrate (Fig. 1). This approach circumvents the misleading effects resulting from soluble quinone analogues, and allows the contributions of SQs from other respiratory chain elements to be probed. Although a chimeric respiratory chain using SMPs and the purified cytochrome *bd* complex from *Escherichia coli* has been described previously [21], it is unsuitable for SQ studies owing to the very stable and therefore dominant cytochrome *bd* SQ [22, 23]. Addition of the alternative oxidase (AOX) from *Trypanosoma brucei brucei* to SMPs creates ‘AOX-SMPs’, containing a quinone-cycling system between complex I and AOX and with NADH:O_2_ turnover sensitive to complex I inhibitors (such as piericidin A and rotenone) and the AOX inhibitor ascofuranone [24, 25]. As no SQ intermediates have been detected by EPR spectroscopy in wild-type AOX [26, 27], incorporation of this terminal oxidase into SMPs represents an intriguing new platform to investigate the SQs that are generated during sustained NADH oxidation by complex I.

## Results

### SMPs and AOX-SMPs are well coupled and sustain a proton-motive force

The variation in SQs observed previously in different preparations of SMPs demands careful characterisation of our membrane system. The SMPs used here (see [28, 29]) are capable of NADH- and succinate-driven ATP synthesis and ATP hydrolysis and are intrinsically well coupled without additional coupling factors, so they are a physiologically relevant system for studying SQ formation. The addition of AOX provides a rapid secondary quinol oxidation route (Fig. 1), such that NADH:O_2_ turnover is capable of exceeding turnover by the canonical respiratory chain in an AOX-concentration dependent manner [24, 25]. Thus, to prevent the substrate from becoming exhausted and ensure turnover during EPR sample preparation, AOX was titrated to a low level, at which the flux through the complex I-AOX pathway matched that through the canonical chain (Additional file 1, Figure S1). Representative catalytic properties for the original SMPs and the rate-matched AOX-SMPs are given in Table 1. In line with previous studies [28, 29], RCR values of 1.6 to 3.0 were typically observed in the absence of AOX, and substantial rates of NAD^+^ reduction (reverse electron transfer, RET, driven by a reduced Q-pool formed by succinate oxidation and a proton-motive force (Δp) formed by ATP hydrolysis) were observed in both cases (Table 1). The value of Δp established by ATP hydrolysis (~ 160 mV, Table 1) was determined by varying the potential of NADH:fumarate oxidoreduction, and matches previously reported values [24, 28]. Therefore, the loss of the RCR in AOX-SMPs does not arise from a loss of coupling, since the Δp supported by ATP hydrolysis is shown to be substantial and near identical to that in the original SMPs.

**Table 1:** Representative kinetic measurements on SMPs and AOX-SMPs. AOX-SMPs were preincubated with antimycin A. For assay details, see “Methods”

	NADH:O_2_ (μmol min^− 1^ mg^− 1^)			Succinate:NAD^+^ (RET) (μmol min^− 1^ mg^− 1^)		Inhibition of NADH oxidation (%)		Δp (mV)
	−Gram	+Gram	RCR	−Gram	+Gram	Piericidin A	Quinol oxidation^a^	
**SMPs**	0.301 ± 0.008	0.626 ± 0.011	2.08 ± 0.05	0.092 ± 0.004	0.009 ± 0.001	98.7 ± 0.01	95.8 ± 0.03	161.4 ± 0.2
**AOX-SMPs**	0.288 ± 0.026	0.360 ± 0.028	1.25 ± 0.03	0.065 ± 0.004	0.007 ± 0.005	99.2 ± 0.01	97.1 ± 0.01	157.9 ± 1.6

^a^Quinol oxidation was inhibited by antimycin A in SMPs, and antimycin A and ascofuranone in AOX-SMPs

**Fig. 2:**
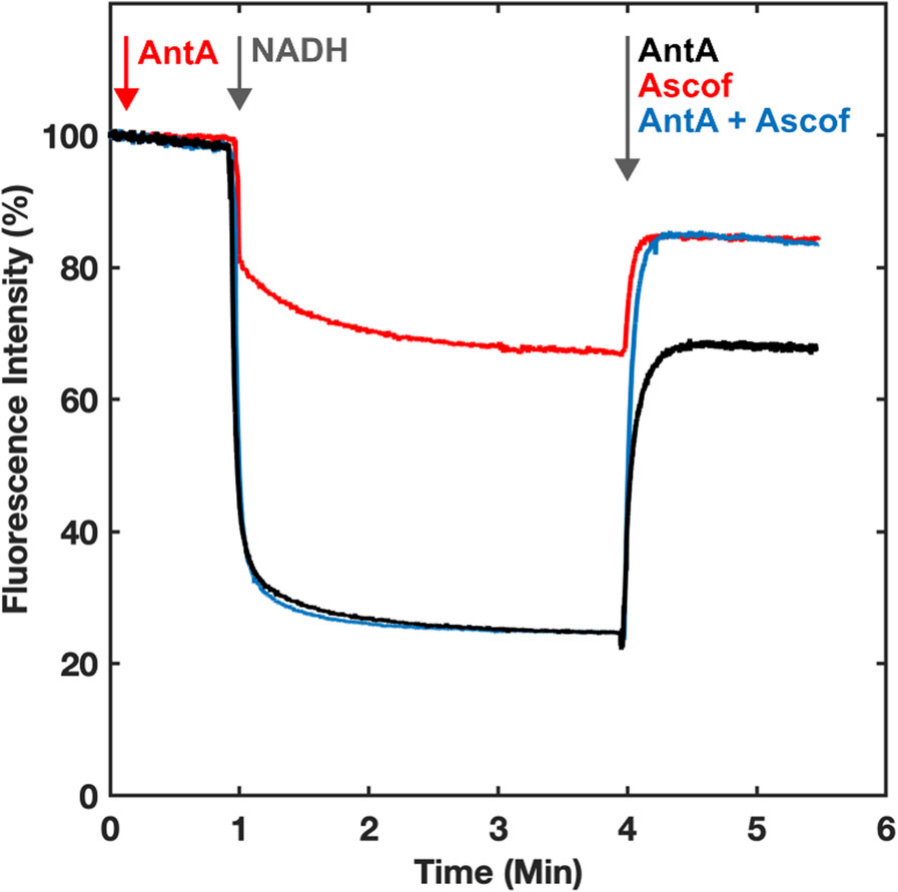
The effect of oxidase pathway inhibitors on quenching ACMA fluorescence in AOX-SMPs. ACMA fluorescence quenching was measured (excitation at 419 nm, emission at 484 nm) during NADH:O_2_ turnover in AOX-SMPs. The rate of coupled NADH oxidation in AOX-SMPs was rate-matched as in Additional file 1, Figure S1. Measurements were performed with constant stirring at 32 °C in a buffer containing 10 mM Tris-SO_4_, 50 mM KCl. In total, 50 μg/mL of SMPs or AOX-SMPs were added to a mixture containing 0.5 μM ACMA and 0.1 μM valinomycin. NADH was added to 500 μM. Turnover was inhibited by addition of antimycin A (2 μM) and/or ascofuranone (2 μM) as indicated

The proton-pumping capacity of AOX-SMPs was further visualised through quenching of ACMA fluorescence during NADH:O_2_ turnover (Fig. 2). Substantial quenching was observed in AOX-SMPs upon addition of NADH. The addition of antimycin A (either before or after NADH addition) causes a partial ablation of the ACMA fluorescence quenching due to continued catalysis and proton pumping by complex I. A combination of antimycin A and the specific AOX inhibitor ascofuranone fully ablates quenching. Following prior incubation with antimycin A, the level of ACMA quenching from complex I turnover is approximately 30–40% of the quenching without any inhibitor, as expected for the rate-matched AOX-SMPs from the established stoichiometry [29]. Quenching was fully sensitive to complex I inhibition by piercidin A, as well as to uncoupling to dissipate Δp (Additional file 2, Figure S2). Levels of ACMA quenching were identical in both SMPs and AOX-SMPs (Additional file 2, Figure S2) in the absence of inhibitors, and ACMA quenching by SMPs and AOX-SMP during ATP hydrolysis matches closely (Additional file 2, Figure S2). This further demonstrates that the coupling of the membrane is retained in the AOX system.

### The *g* ~ 2 EPR signal in the presence of O_2_ in SMPs

**Fig. 3:**
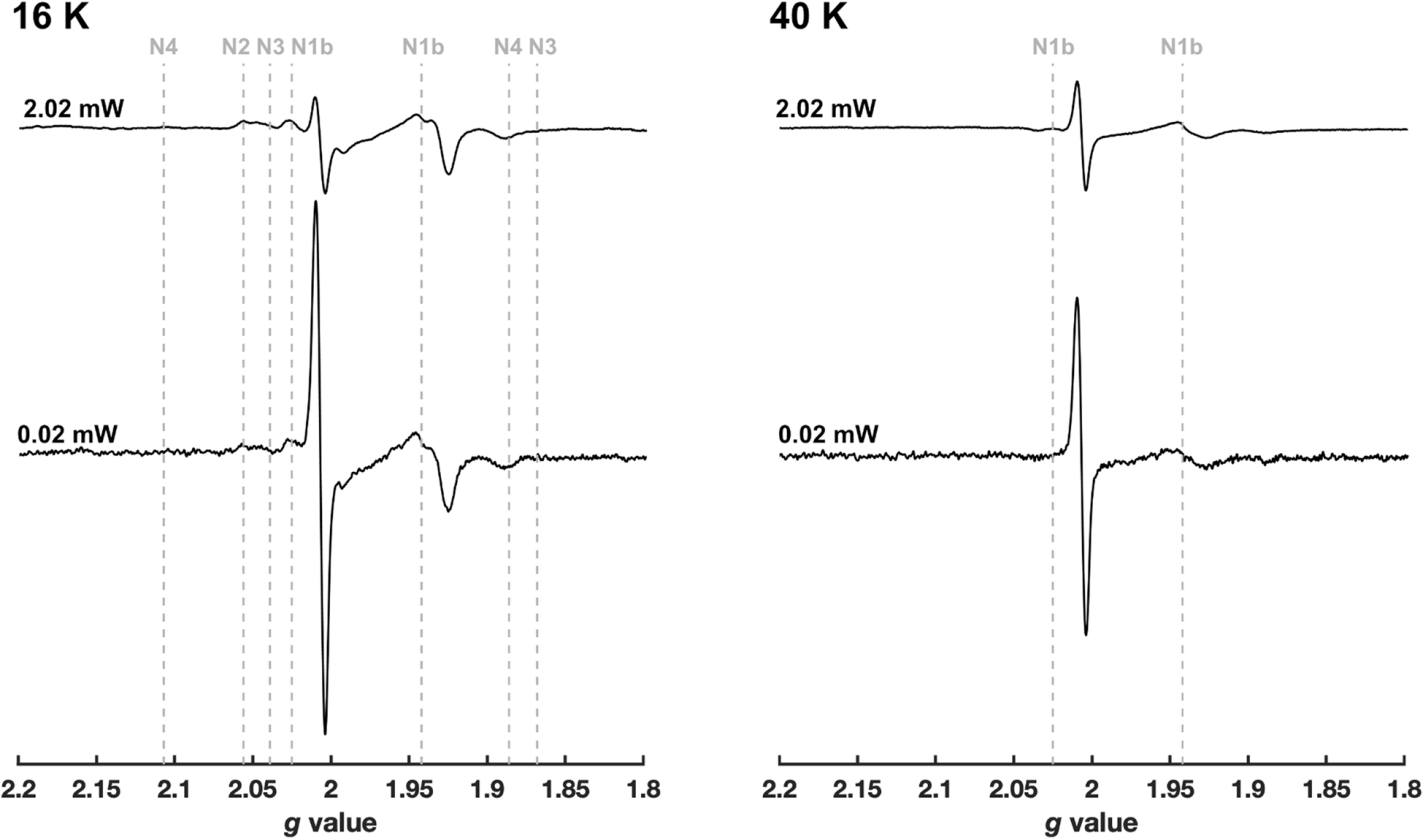
X-Band continuous-wave EPR spectra of oxygen-supplemented SMPs reduced with NADH. The sample was reduced by 15 mM NADH (final concentration) to approximately 25 mg/mL SMPs under continuous O_2_ flow in the absence of any inhibitor. Following mixing, the sample was flash-frozen within 7 s (see “Methods”). All spectra have been normalised by the square root of the microwave power, and both panels are shown at the same relative intensity. Vertical lines show the principle *g* values for the Fe-S clusters of complex I

In agreement with previous work [30], a large radical signal is observed at *g* ~ 2 (Fig. 3) upon addition of NADH to SMPs supplemented with additional O_2_. This signal is almost negligible in SMPs not supplemented with O_2_ (Additional file 3, Figure S3), and it is typically attributed to SQs arising from the respiratory chain complexes. The relatively narrow linewidth (~ 9 G) defines the signal as arising from one or more SQ species, rather than a complex I flavosemiquinone, as this would exhibit a broader linewidth (> 15 G) [31, 32]. The other EPR signals are primarily due to Fe-S clusters in complex I (referred to using the Nx nomenclature) and the other respiratory complexes, which are predominantly reduced during turnover. Due to their faster relaxation compared to the *g* ~ 2 radical signal, which makes the radical signal stand out more at lower power (0.02 mW), the Fe-S signals are best observed at lower temperature (16 K). At higher temperature (40 K), the slowest-relaxing complex I [2Fe-2S] cluster N1b dominates the Fe-S region of the spectrum, alongside the prominent *g* ~ 2 signal. The broad dip in the same region from the oxidised Cu_a_ centre of complex IV (most prominently observed at 16 K owing to the fast relaxation of the Cu_a_ centre) confirms the sustained turnover condition [33] in the presence of O_2_.

### Assignment of the *g* ~ 2 EPR signal through inhibition studies

**Fig. 4:**
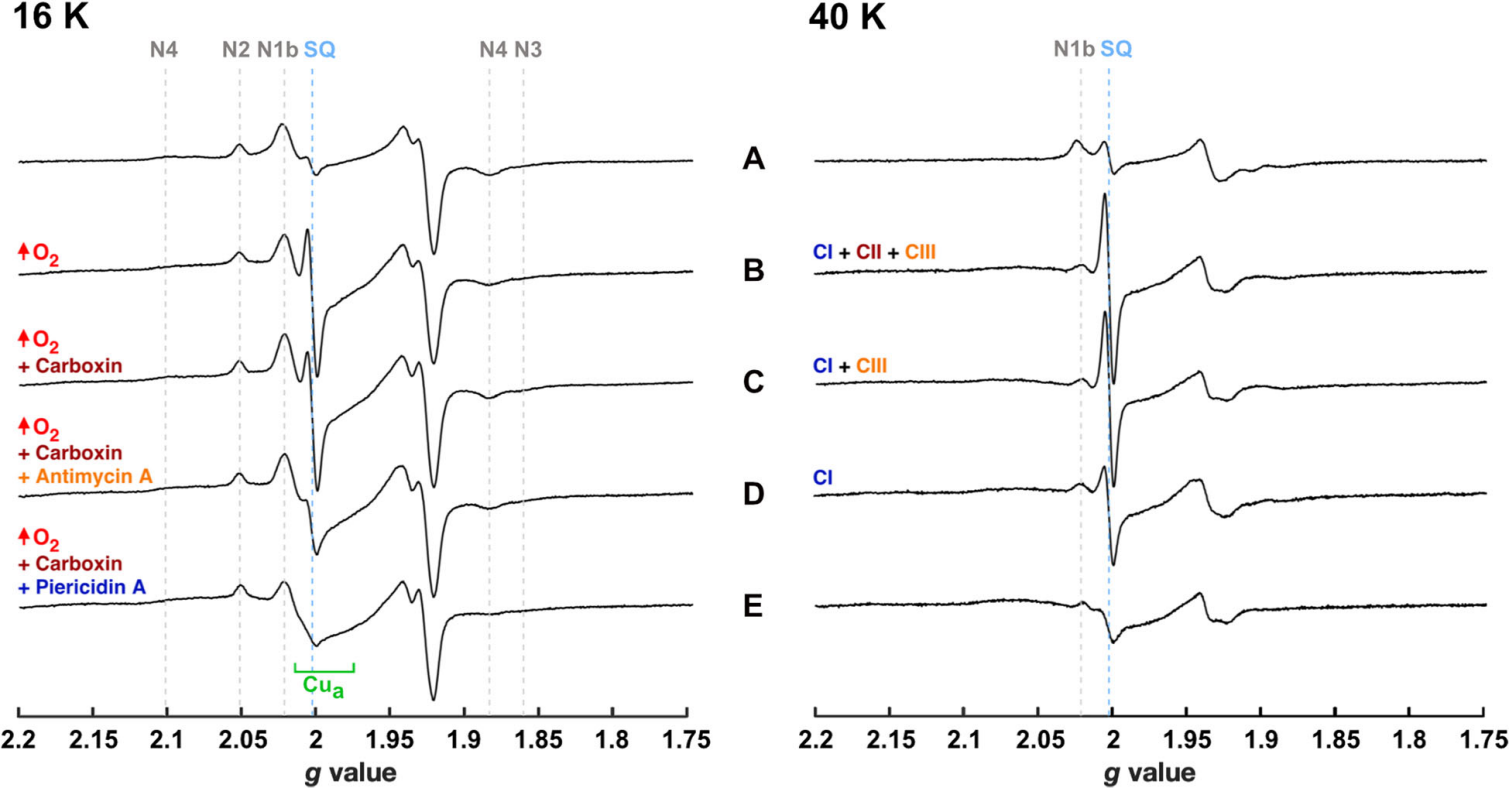
X-band continuous-wave EPR measurements of AOX-SMPs during NADH:O_2_ turnover in the presence of respiratory chain inhibitors. 16 K (left) and 40 K (right) EPR spectra of AOX-SMPs (25 mg/mL SMP protein and 0.01 mg AOX mg^− 1^) reduced by 20 mM NADH in presence of the inhibitors marked and supplemented by additional O_2_ flow where stated. Sample A was prepared aerobically, without additional O_2_. The main respiratory chain enzymes that contribute to the SQ signal at *g* ~ 2 in each sample are indicated on the right-hand panel. Inhibitor concentrations: 100 μM carboxin (complex II Q-site inhibitor), 100 μM antimycin A (complex III Q_i_ site inhibitor),100 μM piericidin A (complex I Q-site inhibitor). EPR measurement conditions: 2.02 mW microwave power

**Fig. 5:**
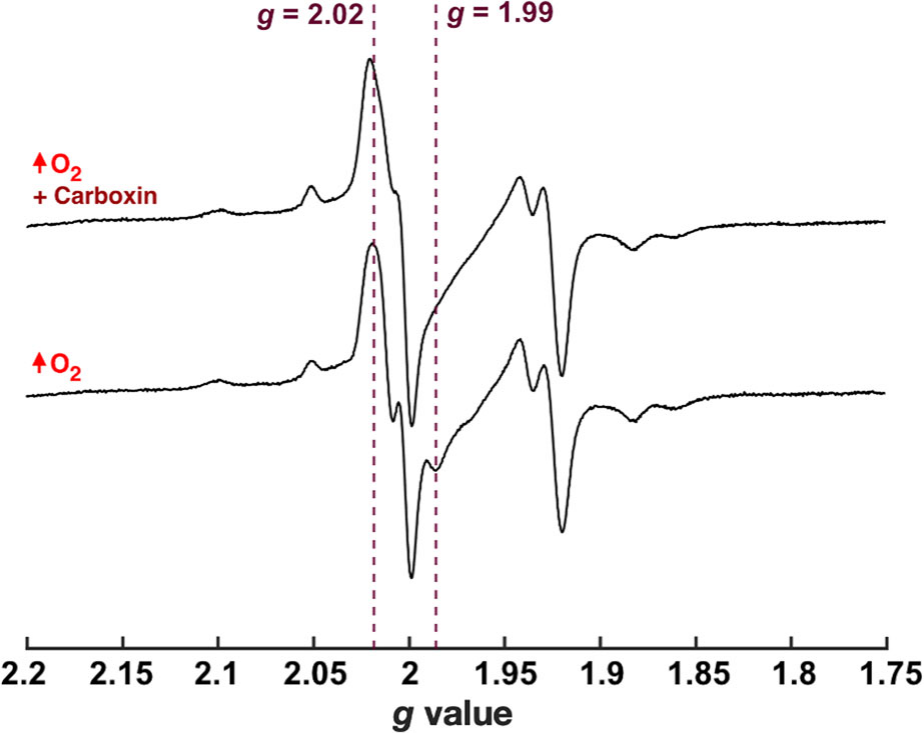
The effect of carboxin on complex II semiquinone signals at 12 K. AOX-SMPs were prepared and measured as in Fig. 4 but at 12 K. Vertical lines indicate *g* values for the SQ-SQ radical pair of complex II as previously reported [34]

To assign the origin of the prominent *g* ~ 2 EPR signal, a series of AOX-SMP samples were prepared containing different inhibitors (Fig. 4). AOX-SMP samples were chosen rather than SMP samples because turnover in the presence of inhibitors can only be sustained by employing the chimeric respiratory chain (Fig. 1). Akin to what is observed for SMPs, the *g* ~ 2 EPR signal increases substantially in intensity in the presence of additional O_2_ (Fig. 4A, B). Note that all spectra in Fig. 4 were recorded at a high microwave power (2.02 mW) to use the (fast-relaxing) Fe-S cluster signals for reference. Carboxin, a complex II inhibitor, ablated a contribution observed most clearly at *g* = 1.99 (more clearly seen at 12 K, see Fig. 5) that results from a well-documented SQ-SQ radical pair in the complex II Q-site [34, 35] and is a consequence of the highly reduced Q-pool during NADH:O_2_ turnover (Fig. 1). However, carboxin had little effect on the overall *g* ~ 2 signal (compare Fig. 4B, C and see Additional file 4, Figure S4 for the dependence of signal intensity on microwave power). Antimycin A, a complex III Q_i_ site inhibitor, diminished the *g* ~ 2 signal intensity substantially (Fig. 4D). The major SQ species present during NADH oxidation (note that complex I turnover continues in the presence of antimycin A, due to the presence of AOX) must therefore arise from complex III. Finally, the complex I specific Q-site inhibitor piericidin A effectively eliminates the *g* ~ 2 signal (Fig. 4E) because it prevents quinone reduction and SQ radicals forming anywhere in the chain. Therefore, the *g* ~ 2 signal comprises (at least) three distinct SQ species: the major contribution is from complex III, there is a lesser contribution from complex II, and a further minor contribution (revealed by comparison of Fig. 4D and E) that may be, but is not proven to be, from complex I.

### Exploiting sustained turnover to investigate the possible complex I SQ

**Fig. 6:**
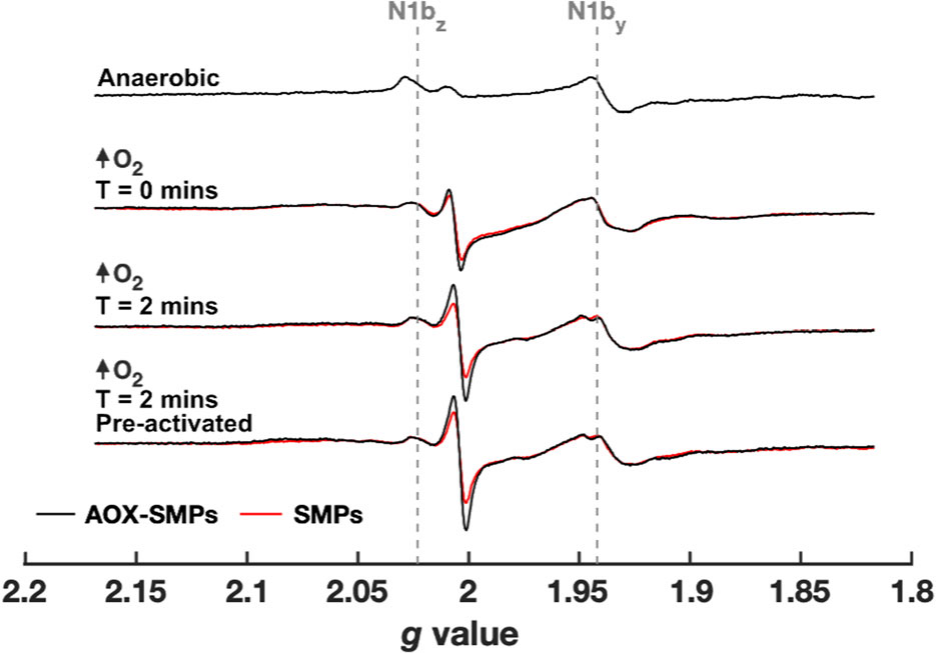
Comparison of SQ EPR signals in antimycin A- and carboxin-inhibited SMPs and AOX-SMPs. X-band EPR spectra of SMPs (red) and AOX-SMPs (black, 0.01 mg AOX mg^− 1^) frozen immediately or following a 2-min incubation with 40 mM NADH under high O_2_ flow. Samples were treated with 100 μM carboxin and 100 μM antimycin A. Pre-activated samples were mixed with 1 mM NADH and incubated at room temperature for 5 min in an aerobic atmosphere prior to EPR sample preparation. The anaerobic AOX-SMP sample was incubated in a Braun UniLab-plus glovebox (O_2_ < 0.5 ppm, N_2_ atmosphere) at room temperature (22 °C) for 1 h prior to addition of 20 mM NADH. The sample was frozen within 30 s of substrate mixing. EPR measurement conditions: 2.02 mW microwave power, 40 K

Being as the AOX-SMP system enables steady-state complex I turnover independently of complex III, we reasoned that any *g* ~ 2 SQ signals from complex I in the presence of NADH (with complexes II and III inhibited) should be retained in AOX-SMPs (complex I is turning over) but not in SMPs (complex I is not turning over). Figure 6 shows that the *g* ~ 2 signal observed is larger in AOX-SMPs compared to SMPs (the increase is small but highly consistent) and that it is amplified over time but does not increase further with prior activation of complex I. The relative increase in the *g* ~ 2 signal in AOX-SMPs over time is thus unlikely to result from activation of complex I and could result from evolution of the oxidation state of the ubiquinone pool. However, the presence of *any g* ~ 2 signal in SMPs that are not undergoing turnover is surprising: complex II/III-inhibited SMPs should emulate the anaerobic condition in which the ubiquinone pool simply becomes fully reduced by NADH (Fig. 6, top spectrum), and therefore the *g* ~ 2 signal intensity is expected to be insignificant. The origin of the *g* ~ 2 signal in Fig. 6, which thus appears to be stabilised or induced by the presence of O_2_, is therefore unclear. Its sensitivity to piericidin A in AOX-SMPs (Fig. 4E) make it tempting to assign it to complex I, but its presence in SMPs inhibited with antimycin A means that downstream effects mediated by changes in the potential of the ubiquinone pool, perhaps in other Q-linked enzymes, cannot be excluded.

### Using hyperfine spectroscopy to distinguish overlapping SQ signals

**Fig. 7:**
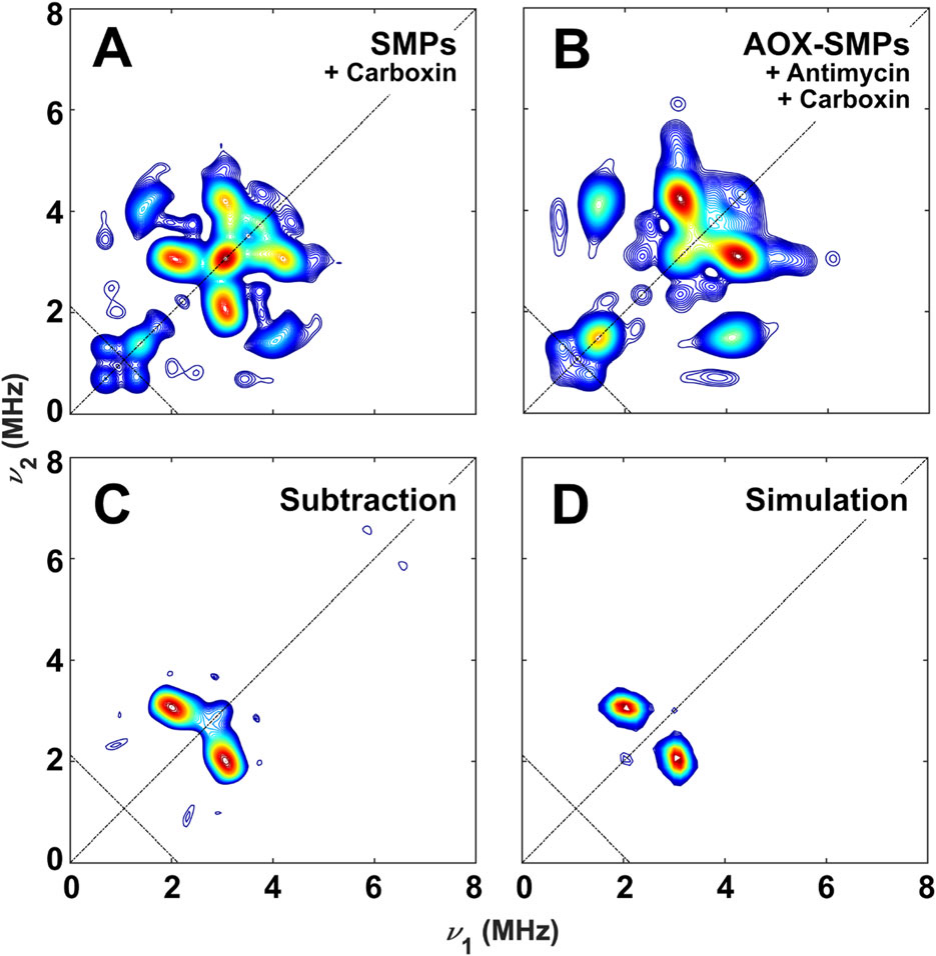
Comparison of ^14^N couplings in HYSCORE spectra of SMPs and AOX-SMPs at 40 K. X-band spectra (**a** and **b**) were recorded at *g* = 2.006 with a four-pulse sequence (π/2–τ–π/2–*t*_1_–π–*t*_2_–π/2–τ–echo) with π/2 = 16 ns, π = 16 ns, τ = 116 ns, shot repetition time 2 ms. The intensity of the stimulated echo (**c**) was normalised to the intensity of the cross peaks at [4.1, 1.6] MHz. Simulations (**d**) were calculated as a single ^14^N nucleus coupled to a single electron spin, with parameters *A*_iso_ = 0.54 MHz, *T* = − 0.2 MHz, *κ* = 0.425 MHz, *η* = 0.18 MHz (see Eq. 3 for calculation of quadrupole coupling parameters). Antidiagonal lines are given for the ^14^N Larmor frequency. Very similar data as in **a** were recorded for AOX-SMPs in the presence of carboxin (Additional file 5, Figure S5); the data shown here are for SMPs because of their higher signal to noise ratio. No strongly coupled ^14^N nuclei were detected in the (−,+) quadrant; for the entire (+,+) quadrant (including the ^1^H region), see Figures S6 and S7 in Additional files 7 and 8, respectively

As X-band CW EPR spectroscopy cannot unambiguously distinguish different SQ species (their *g* values and linewidths are too similar), we employed hyperfine spectroscopy to differentiate the SQs on the basis of the surrounding nuclear spins (^14^N, *I* = 1) coupled to the electron spin. Equivalent but more concentrated (~ 2x) samples than those shown in Fig. 4 were prepared for HYSCORE spectroscopy. The ^14^N regions of the 40 K HYSCORE spectra of carboxin-treated, and carboxin- and antimycin A-treated samples are shown in Fig. 7.

**Fig. 8:**
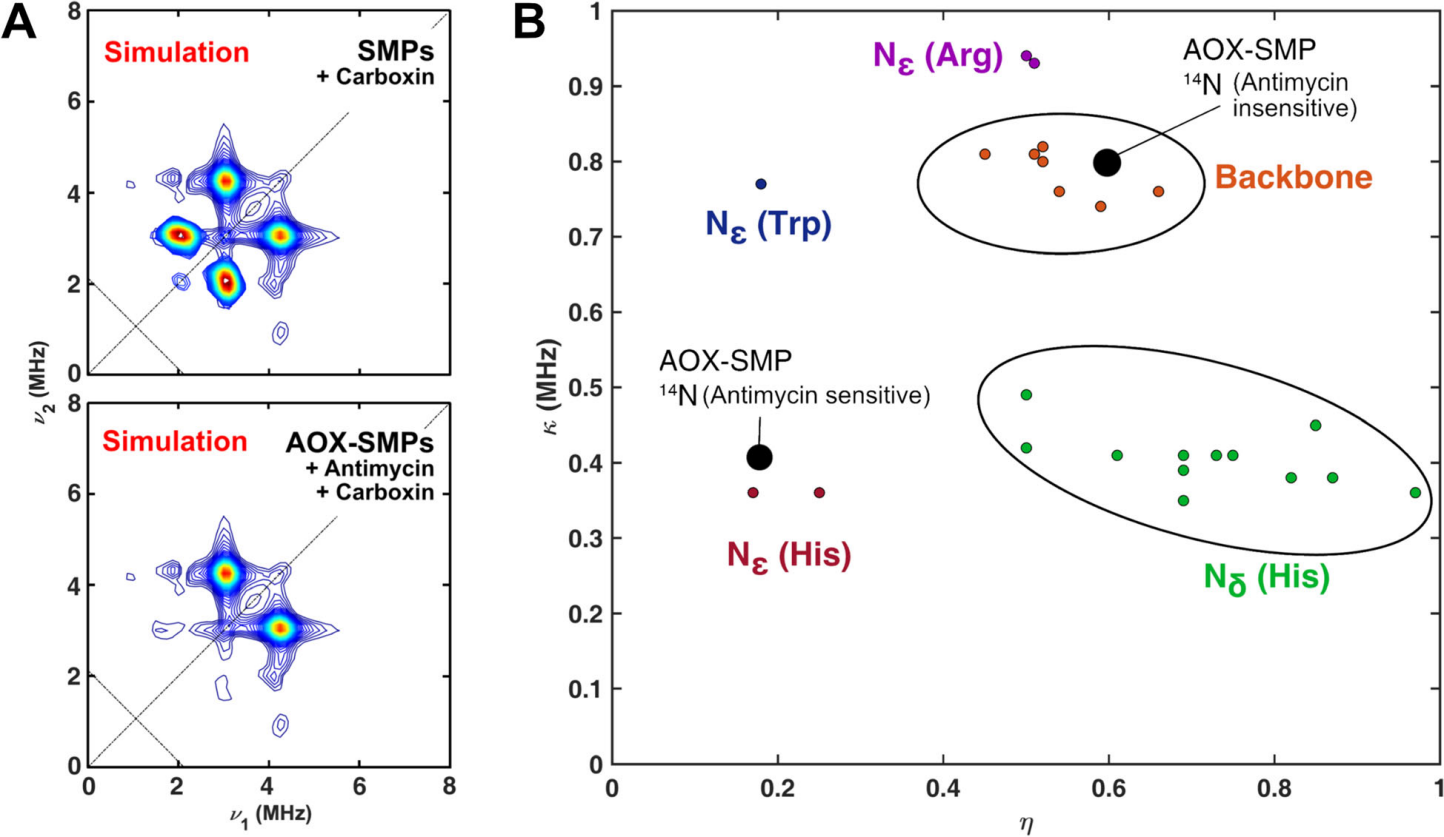
Analysis of ^14^N couplings from antimycin A-inhibited AOX-SMPs: Quadrupole coupling parameters indicate the identity of ^14^N nuclei. **a** Simulated HYSCORE spectrum of SMPs inhibited with carboxin and AOX-SMPs inhibited with carboxin and antimycin. Simulation of major ^14^N cross peaks with *A*_iso_ = 1.00 MHz, *T* = 0.08 MHz, *κ* = 0.8 MHz, *η* = 0.6 MHz (Antimycin insensitive) and *A*_iso_ = 0.54 MHz, *T* = − 0.2 MHz, *κ* = 0.425 MHz, *η* = 0.18 MHz (Antimycin sensitive). ^14^N cross peaks were simulated as two electron spins each coupled to a single ^14^N nucleus. See methods (Eq. 3) for quadrupole coupling parameters. **b** Reported ^14^N quadruple coupling parameters for protein-bound SQs. The two observed sets of ^14^N couplings from AOX-SMP are labelled on the basis of their antimycin A sensitivity (both species are carboxin insensitive). **b** was adapted from [37]

Whereas the spectrum from the complex II-inhibited sample exhibits two sets of ^14^N cross peaks at [3.1, 4.3] MHz and [3.0, 2.1] MHz (Fig. 7a), the latter peaks are absent when both complex II and III are inhibited (Fig. 7b). Indeed, the HYSCORE difference spectrum reveals a single set of ^14^N cross peaks (Fig. 7c) that could be simulated (Fig. 7d) with hyperfine and quadrupolar parameters that are highly consistent with those determined for the strongly H-bonded Q_i_ site SQ radical in complex III isolated from *Rhodobacter sphaeroides* which forms an interaction with the N_ε_ of a histidine (Additional file 6, Table S1) [36]. Therefore, the HYSCORE measurements enable unambiguous assignment of the antimycin A-sensitive SQ species to complex III, consistent with the conclusion from CW measurements (Fig. 4) that the major SQ radical stabilised in SMPs during NADH:O_2_ turnover arises from complex III. The complete simulation of the ^14^N environment of the SQ in carboxin-inhibited SMPs is given in Fig. 8a.

The quadrupole coupling parameters (*κ* and *η*—see Eq. 3) of ^14^N nuclei can be highly informative as to the type of nitrogen interacting with the electron spin (Fig. 8b). The prominent set of cross peaks at [3.1, 4.3] MHz present in both samples could be simulated well with hyperfine and quadrupole parameters that are typical of backbone amide ^14^N (Fig. 8a, see also Additional file 6, Table S1), as revealed by comparison with the ^14^N quadrupole parameters of known protein-bound SQs (Fig. 8b). Given that contributions from the Fe-S clusters (specifically N1b, see ref. [38]) are minimal relative to the contributions from the *g* ~ 2 signal (shown by measurements conducted off-resonance from the *g* ~ 2 SQ signal, see Additional file 7, Figure S6 and Additional file 8, Figure S7), these ^14^N modulations must originate from a SQ radical. It is tempting to attribute the cross peaks to complex I SQ (given that complex II is inhibited in both samples), but their generic spectroscopic signature necessitates caution and a definitive assignment cannot be made.

## Discussion

The AOX-SMPs used here have enabled us, for the first time, to investigate the effects of respiratory chain inhibitors on SQ species formed downstream of complex I, while sustaining complex I turnover with its native ubiquinone substrate. Many previous studies into complex I SQs have refrained from using inhibitors due to emphasis on the requirement for sustained turnover [8, 30, 39]. However, SQs can be stabilised by complexes I, II [35, 40, 41] and III [36, 42, 43], as well as by many other candidate off-pathway enzymes, so inhibitors have a crucial part to play in dissecting their origins. Inhibitors have been combined with NADH:Q_1_ turnover, but Q_1_ and other soluble ubiquinone analogues are known to react at the complex I flavin site through SQ intermediates in potentially misleading side reactions [18]. Our chimeric respiratory chain supports sustained complex I turnover while avoiding these side reactions. Furthermore, the membrane particles used here show substantial proton-pumping capacity (without ‘induced coupling’ by oligomycin or other poorly characterised treatments) as demonstrated by ACMA fluorescence and Δp measurements, and the AOX-SMP system has recently been verified as a physiologically relevant model for oxidative phosphorylation, with substantial rates of ATP synthesis and confirmed proton-pumping stoichiometries [24].

The majority of studies of SQ radicals in SMPs have used deconvolution of power saturation curves to determine the number of SQ species present, along with their relative relaxation rates. However, power saturation curves are prone to overinterpretation, with better fits to a single curve obtained by increasing the number of independently variable parameters [13], and they are unable to localise species to any particular respiratory chain component. Here we have used, for the first time, pulse EPR methods to investigate the origin of SQs in SMPs—methods that were not available when the first investigations were carried out some 40 years ago. HYSCORE measurements, combined with the modular AOX-SMP system, allowed us to spectroscopically isolate the Q_i_ site SQ in mammalian complex III for the first time, providing details of specific nitrogen hydrogen bonding interactions that closely resemble those in its purple bacterial counterpart [44]. Figure 7 shows that the quadrupolar parameters from the H-bonded complex III SQ_i_ are distinctive, strongly supporting our assignment. Recent work on the complex III SQ allows us to further qualify the type of complex III SQ we are observing. First, we consider the recently discovered Q_o_ site metastable SQ [45] to be an unlikely contributor to the complex III *g* = 2.00 signal because Q_o_ site SQs have not been reported under steady-state aerobic conditions in SMPs as employed here [43]. Two populations of SQ are in principle possible in the Q_i_ site, one with a dipolar coupling interaction to the neighbouring oxidised (paramagnetic) heme *b*_H_ and another without magnetic coupling to the reduced (diamagnetic) state of the heme [46]. The magnetically coupled Q_i_ site SQ is not a contributor under the measurement conditions employed here because its enhanced spin-lattice relaxation means that it is only detectable at high temperature (200 K) and microwave power [44, 46]. The slower relaxing uncoupled Q_i_ site SQ on the other hand can be formed during the oxidation of ubiquinol at the Q_i_ site and is fully compatible with the spectroscopic properties of the Q_i_ site semiquinone generated here in SMPs.

Comparison of our observed CW EPR spectra with known spectra from a radical pair in complex II (Fig. 5), and our HYSCORE spectra with known spectra from SQ in the complex III Q_i_ site (Fig. 7), has thus allowed most of the SQ signal observed during NADH oxidation in SMPs to be assigned. Quantification of the SQ signal in the O_2_-rich samples shown in Fig. 4 gave estimated values of ~ 52% occupancy (SQ per N2), decreasing to ~ 41% in the presence of carboxin, and to ~ 15% in the presence of both antimycin A and carboxin, indicating that complex III makes the largest known contribution, followed by complex II. Thus, while the aim of this study was to characterise the SQ(s) present in complex I, only limited and ambiguous evidence has been obtained for these species. A minor, piericidin A-sensitive signal, observed under high O_2_ partial pressures in AOX-SMPs and SMPs in the presence of carboxin and antimycin A may arise from complex I turnover—but it may also arise from any other enzyme linked to the reduced ubiquinone pool. Since this signal (or, at least, a contribution to this signal) is observed in antimycin A-inhibited SMPs (that have no oxidase activity, Fig. 6), complex I turnover is not required for its presence—but nonetheless, it is absent when complex I is reduced by NADH under anoxic conditions (Fig. 6), which should also reduce any other ubiquinone-linked enzyme. Therefore, the results suggest that the signal arises from an interaction with O_2_, under the high O_2_ partial pressures used here to sustain turnover. Non-catalytic oxidation of ubiquinol itself, or of the cofactors in complex I and other enzymes, may allow the slow turnover of complex I, or O_2_ may interact directly with bound quinones in complex I or other enzymes to generate SQ species. The increase of the *g* ~ 2 signal observed in AOX-SMPs (Fig. 6) may result from an increased steady-state concentration of complex I SQs, but this interpretation remains tentative. Alternatively, for example, AOX-SMPs may harbour the increasing product of a catalytic side reaction that either involves AOX or depends strongly on the ubiquinone pool potential. Therefore, we are unable to definitively assign any of the *g* ~ 2 EPR signals observed to complex I.

Based on the demonstrable proton-pumping capacity of the SMP and AOX-SMP preparations used here, we expected that the *g*_z_ component of the N2 cluster should be split by magnetic interactions with complex I SQ (SQ_Nf_). This phenomenon was not observed. Changes in the region of the N2 *g*_z_ component were observed but required prolonged incubation with NADH, and the N2 *g*_z_ signal intensity was not affected (Additional file 9, Figure S8). Furthermore, they were concomitant with a decrease in the complex II S3 signal and so are unlikely to be associated with the N2 cluster of complex I. We also did not observe a profound reduction of SQ signal intensity at 16 K upon addition of uncouplers [8] (Additional file 9, Figure S8). Our results question the relevance of these features for complex I catalysis, although we cannot unambiguously exclude that they only appear under higher Δp values than were achieved here. While our AOX-SMPs show a lower RCR than those reporting to support N2 signal splitting, it is notable that when the NADH:Q_1_ assay system was used, the RCR value dropped to 1.2–1.5 but N2 splitting and the presence of SQ_Nf_ were still reported [11].

Previous investigations of SQs in SMPs have been inconsistent in their reasons for assigning SQs to complex I, rather than to complexes II and III. Early studies (which produced the most convincing SQ signal relative to the Fe-S spectrum of complex I) did not inhibit complex II or III to ablate their SQs [8, 47] so it is likely that the SQ signals they observed were dominated by enzymes downstream of complex I, particularly complex III. Subsequent studies, including those that relied on NADH:Q_1_ reduction, sought to address this issue [10, 11]. A confident evaluation of the origins of the SQs observed by these studies is difficult because complete EPR spectra were not reported; comparisons of relative intensities were precluded, and the effects of the NADH:Q_1_ system on the Fe-S cluster EPR spectrum left unclear. These studies reported that treatment with carboxin, antimycin A and myxothiazol (a complex III Q_o_ site inhibitor) caused a decrease in total SQ concentration of ~ 50% [10], similar to that observed in our AOX-SMP studies. In contrast, they also determined that (even then) complex I SQs were accumulated to ~ 100% occupancy, with a substantial proportion attributed to ‘SQ_Nx_’, an ill-defined additional putative complex I SQ. The surprisingly high occupancy may be attributed to SQ formation at the flavin by the hydrophilic ubiquinone analogue [18]. The remaining SQ signals were reported to be sensitive to canonical complex I inhibitors such as rotenone and piericidin A during NADH:O_2_ turnover. However, complex I inhibitors prevent reduction of the Q-pool, so cannot definitively localise any SQ to complex I. Finally, attempts to identify complex I SQ species in proteoliposomes, a spectroscopically cleaner system, failed to observe more than a minute fraction of the SQs attributed to complex I in SMPs, despite their reasonable catalytic activity and membrane coupling [12, 20]. This behaviour is consistent with our proposal that the majority of SQ species observed in SMPs do not, in fact, arise from complex I.

## Conclusions

Despite substantial effort, there is currently no consensus on the origins of SQs within the mitochondrial membrane. The inconsistency of the EPR data presented over the past 40 years [13] is not least the result of the complicated nature of SMPs, which has precluded unambiguous assignments of observed SQ signals to individual components within the mitochondrial membrane. Here, we combined a chimeric respiratory chain with advanced pulse EPR spectroscopic techniques to unambiguously assign the major SQ species generated during NADH oxidation in SMPs to respiratory complex III, with an additional minor SQ species resulting from complex II. We conclude that in earlier studies these complex II and III SQs were sometimes mistakenly assigned to complex I, in the absence of the opportunity to conduct, at the time, controlled inhibition studies, pulse EPR measurements, or focused investigations with the native substrate. Although we observed a piericidin A-sensitive SQ signal, which is amplified in the presence of O_2_ and in AOX-SMPs relative to SMPs, and which increases with prolonged turnover, we cannot definitively assign this *g* ~ 2 signal to complex I. The future of this work now lies in isolating any complex I SQ from other *g* ~ 2 signals, to infer mechanistic information on the role of ubiquinone in the complex I energy coupling reaction from its local magnetic environment. This will necessitate conclusive assignment of the SQ signal in antimycin A- and carboxin-inhibited AOX-SMPs to complex I by using a spectroscopically clean system, such as complex I-AOX containing proteoliposomes.

## Methods

### Preparation of AOX-SMPs and EPR samples

SMPs were prepared using a protocol adapted from [28]. Twenty millilitres of mitochondria (~ 50 mg/mL) were defrosted at 4 °C and divided equally into four 50-mL centrifuge tubes. The volume of each sample was made up to 45 mL with SMP buffer (250 mM sucrose, 10 mM Tris-SO_4_ (pH 7.0)), and the mitochondria were centrifuged at 16,000×*g* (16 min, 4 °C). The resulting pellets were combined and homogenised in 40 mL of SMP buffer. The pH of the homogenised mitochondria suspension was adjusted to pH 9.0 with 2.5 M Tris at 4 °C and allowed to stand for 10 min, diluted to 180 mL with SMP buffer, and centrifuged at 48,000×*g* (20 min, 4 °C). The mitochondria were then washed twice more in SMP buffer and collected by centrifugation at 16,000×*g* (16 min, 4 °C). In total, 15 mM MgSO_4_ was added and each 45-mL sample was sonicated at 130 W for a total of 2.5 min (15 s on, 60 s off). The sonicated mixture was centrifuged at 36,000×*g* (20 min, 4 °C) then the supernatant was centrifuged at 120,000×*g* for 30 min to collect the SMPs. The final pellets were suspended and homogenised in approximately 2 mL of SMP buffer, and 100 μL aliquots at 20–30 mg/mL were frozen at − 20 °C. Protein concentrations were determined using the test-tube variation of the Pierce bicinchoninic acid (BCA) protein assay kit (Thermo Fisher Scientific, UK).

The alternative oxidase (AOX) from *Trypanosoma brucei brucei* was prepared and incorporated into SMP membranes as described previously [24, 25]. A 100-μL sample of SMPs was incubated with the desired quantity of AOX (from a 3–5 mg/mL stock) on ice for at least 30 min. Inhibitors were added from DMSO stocks to final concentrations of 100 μM (with the percentage of DMSO kept constant in order to avoid erroneous interpretation of solvent-induced radical scavenging [48]). A total of 100 μL of sample was transferred to a 4.0 mm (O.D.) quartz EPR tube (Wilmad) and incubated at room temperature for 5 min. EPR samples were prepared at 20–25 mg/mL of total SMP protein (~ 2.0–2.5 μM complex I based on approximately 10% complex I content in SMPs [28]). To ensure turnover was maintained until freezing, O_2_ was bubbled through the sample in the EPR tube: samples were placed under O_2_ flow for 30 s, NADH added to approximately 20 mM, and the samples were then mixed under O_2_ flow for 5 s before being frozen by rapid immersion in a cold bath (dry ice/acetone) and transferred to liquid N_2_ for storage.

### NADH:O_2_ activity assays

Kinetic assays were performed with a temperature-controlled Agilent Cary 100 UV-Vis spectrophotometer by following the absorbance of NADH at 340 nm (Δε_340_ = 6.22 mM^− 1^ cm^− 1^). SMP activity assays were performed in SMP buffer (250 mM sucrose, 10 mM Tris-SO_4_ (pH 7.0)) at 32 °C using 30–50 μg/mL SMPs and initiated by 200 μM NADH. Uncouplers (carbonyl cyanide 3-chlorophenylhydrazone (CCCP) or gramicidin, a mixture of gramicidin A, B, C, and D) were added from DMSO stock solutions to 2 μM or 1 μM, respectively. The rate of NADH oxidation in the presence and absence of uncoupler was used to calculate the respiratory control ratio (RCR). Piericidin A, antimycin A, or ascofuranone were added from DMSO stock solutions at 2 μM to inhibit NADH:O_2_ turnover.

### Succinate:NAD^+^ activity assays

Reverse electron transfer (RET) assays followed the reduction of NAD^+^ by SMPs by monitoring the absorbance at 340 nm. A total of 50–100 μg/mL SMPs were added to SMP buffer at 32 °C. Forward electron transfer was inhibited with 2 μM antimycin A (SMPs) or by anaerobic preparation (AOX-SMPs). In total, 1 mM NAD^+^ was added and the assay mixture incubated for 1 min. Then 1 mM MgATP and 10 mM succinate were added to initiate RET. The rate of RET was determined as the maximum rate following succinate addition. Gramicidin (5 μg/mL) or piericidin A (2 μM) was added to inhibit the RET reaction.

### ACMA proton-pumping assays

Proton-pumping assays were performed using an RF-5301PC spectrofluorometer (Shimadzu) and the fluorescent dye 9-Amino-6-Chloro-2-Methoxyacridine (ACMA). The 50 μg/mL SMPs/AOX-SMPs were added to a fluorescence cuvette containing ACMA buffer (10 mM Tris-SO_4_, 50 mM KCl (pH 7.5)) at 32 °C. ACMA was added at 0.5 μM from a DMSO stock solution. Valinomycin was added at 0.1 μM to the buffer solution to diminish the membrane potential (Δψ) contribution of Δp, allowing ΔpH to build to dominate Δp. Proton pumping was initiated by the addition of 500 μM NADH or 1 mM MgATP. ACMA fluorescence was monitored using an excitation wavelength of 419 nm and an emission wavelength of 484 nm. Proton gradients were dissipated by addition of piericidin A (2 μM), antimycin A (2 μM), asocfuranone (2 μM), alamethicin (15 μg/mL), or oligomycin (4 μM).

### Measurement of Δp in SMPs and AOX-SMPs

Δp was measured by varying the potential of NADH:fumarate oxidoreduction during ATP hydrolysis as described previously [28]. Rates were measured in triplicate. The 100 μg/mL SMPs or AOX-SMPs in SMP buffer were treated with 2 μM antimycin A (SMPs) or incubated anaerobically (AOX-SMPs [24]) to inhibit respiration before the addition of 1 mM MgATP. Then, catalysis was initiated by addition of NAD^+^ (1 mM), NADH (100 μM), succinate (500 μM), and variable concentrations of fumarate (0.025 to 40 mM). NADH concentrations were monitored at 340 nm. Δp can be determined by using the different fumarate concentrations to set different potentials via the NAD^+^ (− 0.335 V) and fumarate (0.020 V) potentials, as per Eq. 1:

1 $$ \Delta  E=-0.335+0.020-\frac{RT}{2F}\ln \frac{\left\{\left[\mathrm{NADH}\right]\left[\mathrm{Fumarate}\right]\right\}}{\left\{\left[{\mathrm{NAD}}^{+}\right]\left[\mathrm{Succinate}\right]\right\}}. $$

The potential of zero net rate of catalysis, −2Δ*E* = 4Δp (because complex I pumps 4 protons per two electrons), therefore defines Δp [28].

### EPR measurements

EPR measurements were performed using an X/Q-band Bruker Elexsys E580 Spectrometer (Bruker BioSpin GmbH, Germany) equipped with a closed-cycle cryostat (Cryogenic Ltd., UK) and X-band split-ring resonator module (ER 4118X-MD5), or a Bruker EMXMicro spectrometer with a helium flow cryostat (Oxford Instruments, UK). All measurements were performed at X-band (9.7 GHz). The magnetic field was calibrated at room temperature with a Bruker strong pitch standard (*g* = 2.0028). Baseline spectra of the empty resonator, of samples containing only buffer, as well as of oxidised complex I were found to be identical; all the CW spectra presented have been baseline subtracted. Continuous-wave EPR measurement conditions were 100 kHz modulation frequency, 7 G modulation amplitude; other measurement conditions are given in figure legends.

### EPR simulations

All spectral simulations were performed using the EasySpin package for MATLAB [49, 50]. To simulate HYSCORE spectra, ^14^N parameters were determined according to the spin Hamiltonian for an electron spin (*S* = ½) coupled to a nuclear spin with *I* = 1, with the energy terms representing the electron Zeeman, nuclear Zeeman, hyperfine coupling, and quadrupole coupling, respectively:

2 $$ {\mathcal{H}}_0=\frac{\beta_eg{\boldsymbol{B}}_0\boldsymbol{S}}{\mathrm{\hslash}}-\frac{\beta_n{g}_n{\boldsymbol{B}}_0\boldsymbol{I}}{\mathrm{\hslash}}+ SAI+ IQI, $$

where *β*_*e*_ is the Bohr magneton, *β*_*n*_ is the nuclear magneton, *S* is the electron spin operator (with *S* = ½), *I* is the nuclear spin operator (with *I* = 1 for ^14^N), *g* is the *g* factor (for the semiquinone taken to be isotropic with *g* = 2.006), *g*_*n*_ is the nuclear *g* factor of ^14^N, *B*_0_ is the static magnetic field vector. *A* is the hyperfine tensor, with principal components of the diagonalized matrix *A* = *A*_x,y,z_ = *A*_iso_ + *T*, where *T* = [−*T*, −*T*, 2 *T*] in axial symmetry, as assumed here. *Q* is the nuclear quadrupole tensor, for *I* = 1:

3 $$ \mathrm{Q}=\kappa \left(\begin{array}{ccc}-\left(1-\eta \right)& & \\ {}& -\left(1+\eta \right)& \\ {}& & 2\end{array}\right), $$

where *κ* = $\frac{e^2 qQ}{4\hslash }$ and *η* are the asymmetry parameter 0 < *η* < 1, with 0 designating an axial symmetry and 1 a rhombic symmetry.

Although ^14^N nuclei can produce up to 18 cross peaks, the double quantum (dq_±_, dq_∓_) transitions dominate powder HYSCORE spectra here (as is often the case), giving rise to a single pronounced pair of cross peaks. These cross peaks were simulated by varying the values of the hyperfine parameters, *A*_iso_ and *T*, and the quadrupole components, *κ* and *η*. *A* was estimated from the HYSCORE frequencies of the double quantum transitions using Eq. 4:

4 $$ A=\frac{{\nu_{dq+}}^2-{\nu_{dq-}}^2}{8_{\nu I}}, $$

where *ν*_*I*_ is the nuclear Zeeman frequency. Approximate values of the quadrupole coupling parameters were determined using Eq. 5:

5 $$ {\nu}_{dq\pm }=2{\left[{\nu}_{eff\pm }+{\kappa}^2\left(3+{\eta}^2\right)\right]}^{\frac{1}{2}}, $$

where *ν*_*eff*_ = | *ν*_*I*_ ± *A*/2|, then refined further to better fit the experimental data.

## Supplementary information

Additional file 1. Adjusting the rate of NADH:O_2_ turnover in AOX-SMPs. **Figure S1.** Rate matching of AOX-SMPs to SMP NADH:O_2_ turnover.

Additional file 2. Comparison of the membrane integrity in SMPs and AOX-SMPs. **Figure S2.** Comparison of ACMA quenching in SMPs and AOX-SMPs during NADH oxidation and ATP hydrolysis.

Additional file 3. Comparison of EPR signals of reduced SMPs and isolated complex I. **Figure S3.** X-band CW EPR spectra of reduced SMPs and isolated complex I.

Additional file 4. The microwave power dependence of the g ~ 2 EPR signal in the presence of various inhibitors. **Figure S4.** Power saturation and relative inhibitor effect on semiquinone signal intensity at 40 K.

Additional file 5. Antimycin A-sensitive ^14^N signal in AOX-SMPs. **Figure S5.** HYSCORE spectrum and subtraction of carboxin inhibited AOX-SMPs.

Additional file 6. Summary of the parameters used to simulate ^14^N HYSCORE spectra. **Table S1.** Hyperfine and quadrupole parameters for ^14^N interactions in SMPs and AOX-SMPs.

Additional file 7. HYSCORE spectra of AOX-SMPs at different field positions. **Figure S6.** Echo-detected field sweep and HYSCORE spectroscopy of oxygen supplemented carboxin- and antimycin A-treated AOX-SMPs.

Additional file 8. Complete HYSCORE spectrum of the g ~ 2 signal in complex II inhibited SMPs. **Figure S7.** Echo-detected field sweep and HYSCORE spectroscopy of oxygen supplemented SMPs treated with carboxin.

Additional file 9. Investigating the ‘split N2’ signal. **Figure S8.** The effect of uncouplers on the semiquinone EPR signal in uninhibited SMPs undergoing NADH:O_2_ turnover.

## Abbreviations

AOX: Alternative oxidase
EPR: Electron paramagnetic resonance
Fe-S: Iron-sulphur cluster
HYSCORE: Hyperfine sublevel correlation
RCR: Respiratory control ratio
SMP: Submitochondrial particles
SQ: Semiquinone
