# Supplementary material for: Using a chimeric respiratory chain and EPR spectroscopy to determine the origin of semiquinone species previously assigned to mitochondrial complex I
Source: BMC Biol. 2020 May 20;18:54. doi: 10.1186/s12915-020-00768-6 (PMC7238650; doi:10.1186/s12915-020-00768-6)
Supplement: Supplementary file 1 — Adjusting the rate of NADH:O2 turnover in AOX-SMPs. Figure S1. Rate matching of AOX-SMPs to SMP NADH:O2 turnover. [file 12915_2020_768_MOESM1_ESM.docx]

1. **Adjusting the rate of NADH:O_2_ turnover in AOX-SMPs**

As the rate of NADH:O_2_ turnover in AOX-SMPs can exceed that of the natural respiratory chain, rate-matching experiments of AOX-SMPs and SMPs were performed to ensure that the substrate is not exhausted in AOX-SMPs on the timescale of the EPR sample preparation:


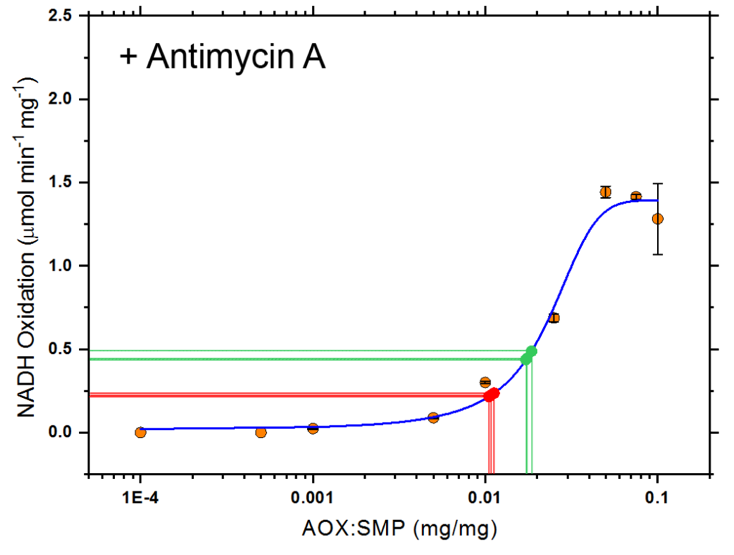


**Figure S1: Rate matching of AOX-SMPs to SMP NADH:O_2_ turnover.** SMPs were incubated with AOX at the ratios specified. Activity assays were performed with 20 µg/mL SMP and 200 µM NADH. Antimycin A (2 µM) was added to the assay mixture to inhibit electron flux through the canonical electron transport chain. The triplicate red lines indicate the amount of AOX required to match the rate of uninhibited SMPs in this experiment. The green lines indicate the amount of AOX required to match the rate of uncoupled (+ 4 μM CCCP) SMPs. Error bars indicate standard deviation of triplicate measurements.
