# Supplementary material for: Using a chimeric respiratory chain and EPR spectroscopy to determine the origin of semiquinone species previously assigned to mitochondrial complex I
Source: BMC Biol. 2020 May 20;18:54. doi: 10.1186/s12915-020-00768-6 (PMC7238650; doi:10.1186/s12915-020-00768-6)
Supplement: Supplementary file 2 — Comparison of the membrane integrity in SMPs and AOX-SMPs. Figure S2. Comparison of ACMA quenching in SMPs and AOX-SMPs during NADH oxidation and ATP hydrolysis. [file 12915_2020_768_MOESM2_ESM.docx]

1. **Comparison of the membrane integrity in SMPs and AOX-SMPs**

The experiments below complement Figure 2 in the main text. SMPs and AOX-SMPs exhibited the same levels of ACMA quenching upon addition of NADH, both in the absence and presence of inhibitors. Moreover, ACMA quenching by SMPs and AOX-SMP during ATP hydrolysis matches closely.

**
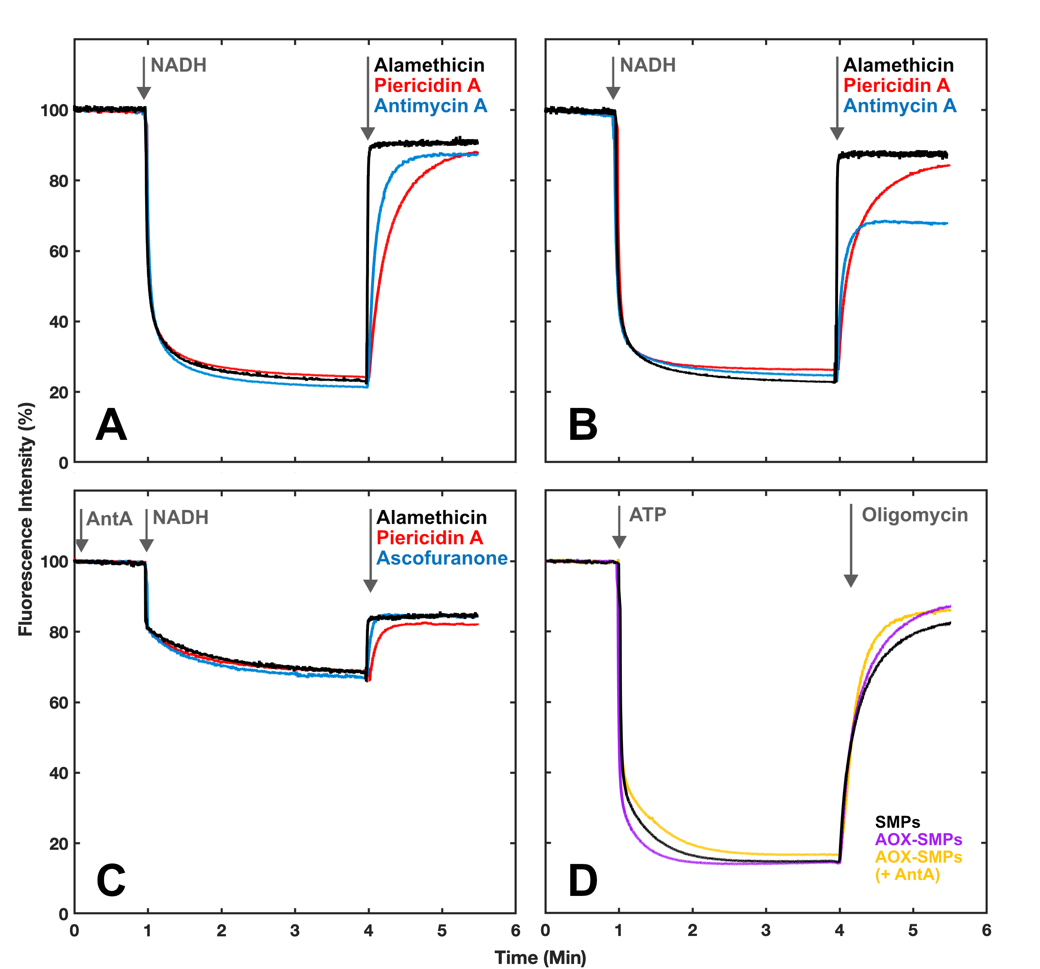
**

**Figure S2: Comparison of ACMA quenching in SMPs and AOX-SMPs during NADH oxidation and ATP hydrolysis.** (A-C) ACMA fluorescence quenching was measured during NADH:O_2_ turnover in (A) SMPs, (B) AOX-SMPs, and (C) AOX-SMPs pre-treated with 2 µM antimycin A. (D) ACMA fluorescence quenching measured during ATP hydrolysis in SMPs (black line) AOX-SMPs (purple line), and AOX-SMPs pre-treated with 2 µM antimycin A (yellow line). Measurements were performed with constant stirring at 32 ºC in 10 mM Tris-SO_4_, 50 mM KCl (pH 7.5). 0.5 µM ACMA and 0.1 µM valinomycin were added. For ATP hydrolysis measurements, 1 mM MgSO_4_ was also present. All measurements contained 50 µg/mL SMP protein. Where present, AOX was added in at 0.01 mg AOX mg^-1^. Turnover was initiated with 500 µM NADH or 1 mM ATP. Fluorescence recovery was achieved by addition of the pore forming uncoupler, alamethicin (15 µg/mL), respiratory chain inhibitors (piericidin A, antimycin A or ascofuranone - 2 µM) or oligomycin (4 µM).
