# Supplementary material for: Using a chimeric respiratory chain and EPR spectroscopy to determine the origin of semiquinone species previously assigned to mitochondrial complex I
Source: BMC Biol. 2020 May 20;18:54. doi: 10.1186/s12915-020-00768-6 (PMC7238650; doi:10.1186/s12915-020-00768-6)
Supplement: Supplementary file 3 — Comparison of EPR signals of reduced SMPs and isolated complex I. Figure S3. X-band CW EPR spectra of reduced SMPs and isolated complex I. [file 12915_2020_768_MOESM3_ESM.docx]

1. **Comparison of EPR signals of reduced SMPs and isolated complex I**

The EPR spectra in the figure below shows that most of the EPR signals in SMPs originate from complex I, but that a contribution from complex II is significant.


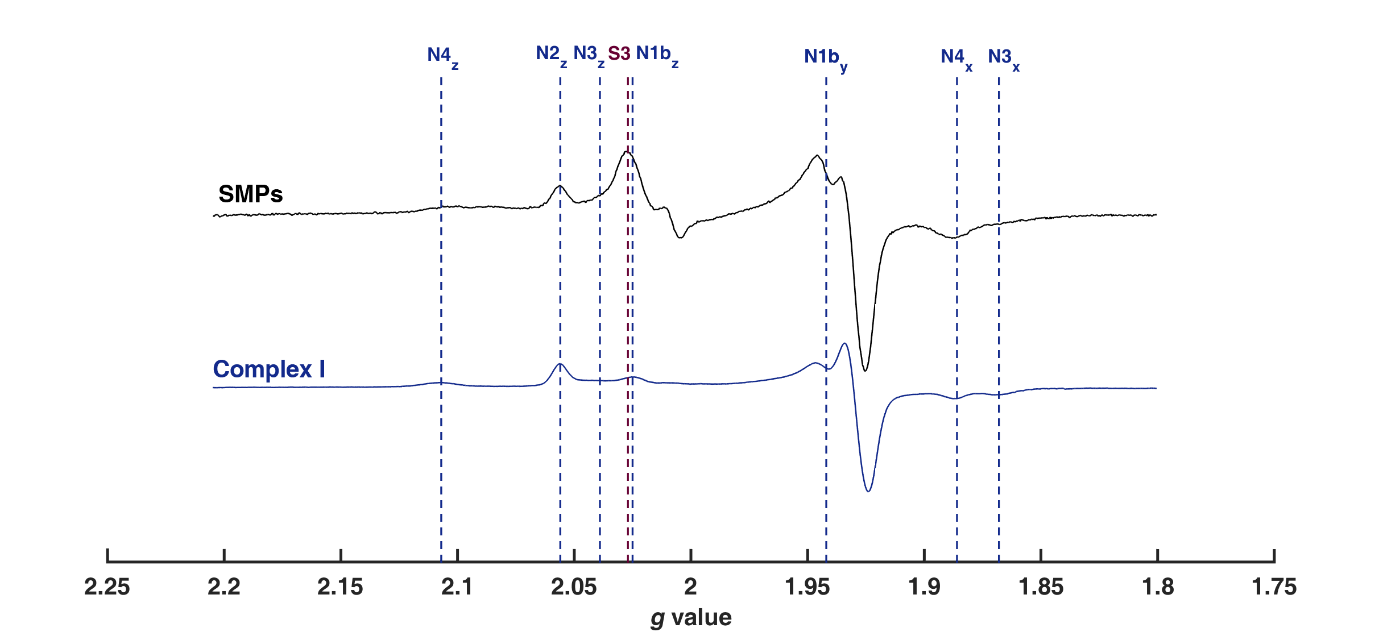


**Figure S3: X-band CW EPR spectra of reduced SMPs and isolated complex I.** SMPs (25 mg/mL) and complex I (25 mg/mL) were reduced with 15 mM NADH under aerobic conditions (atmospheric O_2_). Measurements were carried out at 16 K with 2.02 mW microwave power and 7 G modulation amplitude. Vertical blue lines correspond to the *g* values of the Fe-S clusters in complex I. Species not arising from complex I are indicated in maroon (S3 of complex II at *g* = 2.027). Spectra are normalised to the height of the N2 *g*_z_ signal (*g* = 2.054).
