# Supplementary material for: Using a chimeric respiratory chain and EPR spectroscopy to determine the origin of semiquinone species previously assigned to mitochondrial complex I
Source: BMC Biol. 2020 May 20;18:54. doi: 10.1186/s12915-020-00768-6 (PMC7238650; doi:10.1186/s12915-020-00768-6)
Supplement: Supplementary file 4 — The microwave power dependence of the g ~ 2 EPR signal in the presence of various inhibitors. Figure S4. Power saturation and relative inhibitor effect on semiquinone signal intensity at 40 K. [file 12915_2020_768_MOESM4_ESM.docx]

1. **The microwave power dependence of the *g* ~2 EPR signal in the presence of various inhibitors**

A comparison of the dependence on mw power (through power saturation curves) of the *g* ~ 2 EPR signals is examined below, lending further evidence to the existence of different SQ species in the presence of different inhibitors. Intensities in the power saturation panel (top) are not normalised by microwave power. EPR spectra in the lower 4 panels are normalised to the square root of the microwave power to show the intensity ratio to the N1b (*g* = 1.94) signal, and therefore do not correspond directly to the intensities in the power saturation curves in the top panel.

**Figure S4 (next page): Power saturation and relative inhibitor effect on semiquinone signal intensity at 40 K.** (Top) Power saturation of the semiquinone signal in AOX-SMPs with varying inhibitor and oxygen treatments. Signal intensity was monitored and fit with the equation: *I* = (*c*(√P/(1+P/P_1/2_)*^b^*^/2^), In which *I* is the measured intensity of the EPR signal, *c* is a scaling factor, P is the microwave power of the experiment, *b* is the homogeneity of the saturation and P_1/2_ is the incident microwave power at which the first derivative intensity is half of its saturated value. All samples were treated as a single semiquinone species with the following values: (+ carboxin) *c* = 36.36, P_1/2_ = 0.19 μW, *b* = 0.85. (↑O_2_) *c* = 89.98, P_1/2_ = 0.57 μW, *b* = 0.81. (↑O_2_, + carboxin) *c* = 72.66, P_1/2_ = 0.78 μW, *b* = 0.81. (↑O_2_, + carboxin, + antimycin A) *c* = 36.06, P_1/2_ = 0.17 μW, *b* = 0.67. (Bottom) EPR spectra from which panel A was derived: the relative effect of inhibitors on the semiquinone signal intensity at 40 K. Sample preparation and measurement conditions as in Figure 3, with the power varied to show saturation effects. To enable easy comparison of the relative intensities of N1b and the SQ signal, spectra are normalised to the square root of the microwave power. All panels are shown on the same scale for comparison purposes.

**
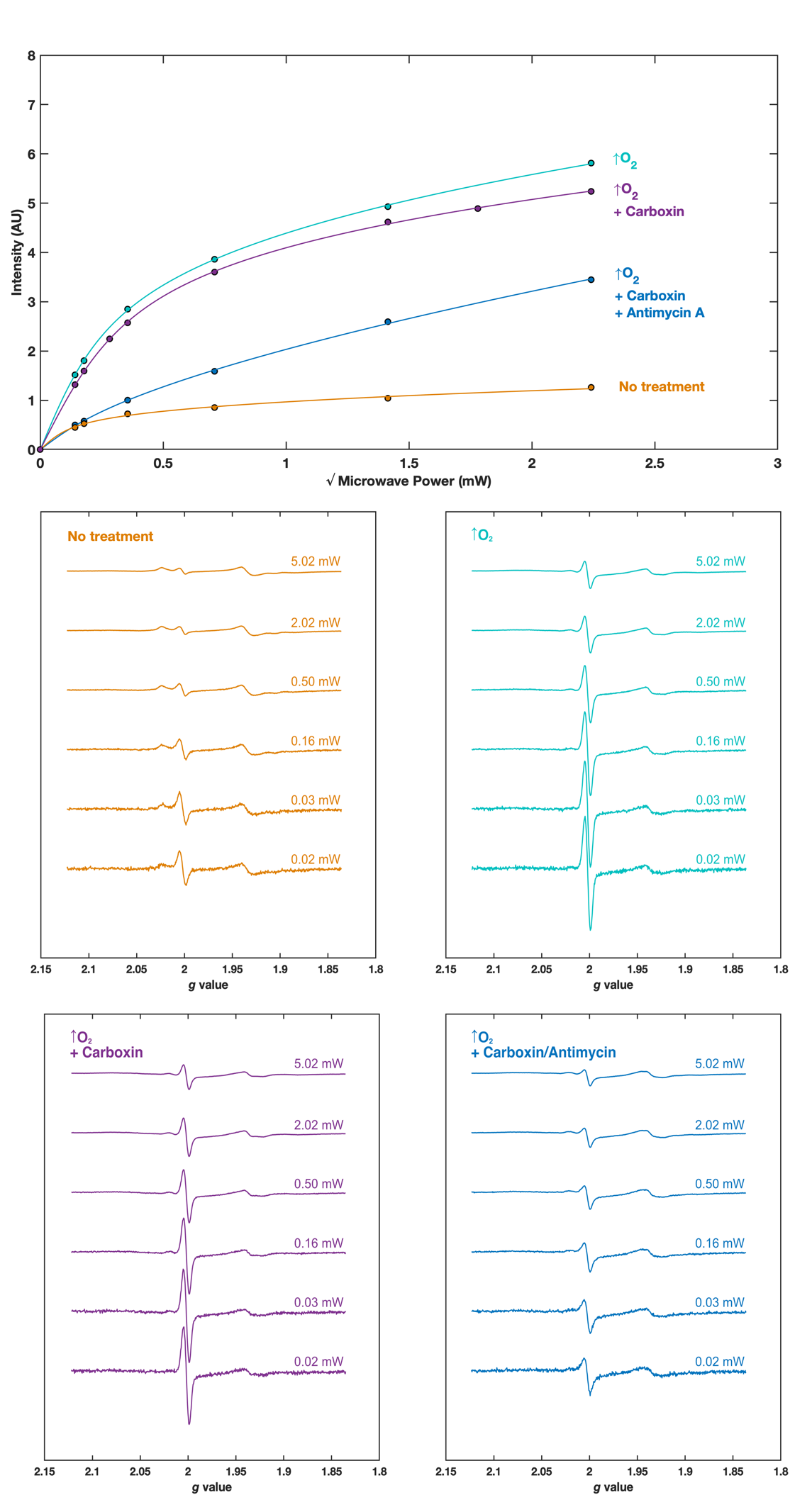
**
