# Supplementary material for: Using a chimeric respiratory chain and EPR spectroscopy to determine the origin of semiquinone species previously assigned to mitochondrial complex I
Source: BMC Biol. 2020 May 20;18:54. doi: 10.1186/s12915-020-00768-6 (PMC7238650; doi:10.1186/s12915-020-00768-6)
Supplement: Supplementary file 5 — Antimycin A-sensitive 14N signal in AOX-SMPs. Figure S5. HYSCORE spectrum and subtraction of carboxin inhibited AOX-SMPs. [file 12915_2020_768_MOESM5_ESM.docx]

1. **Antimycin A-sensitive ^14^N signal in AOX-SMPs**

The same antimycin A-sensitive ^14^N signals were observed in both SMPs (see Figure 7) and AOX-SMPs (see below).

**
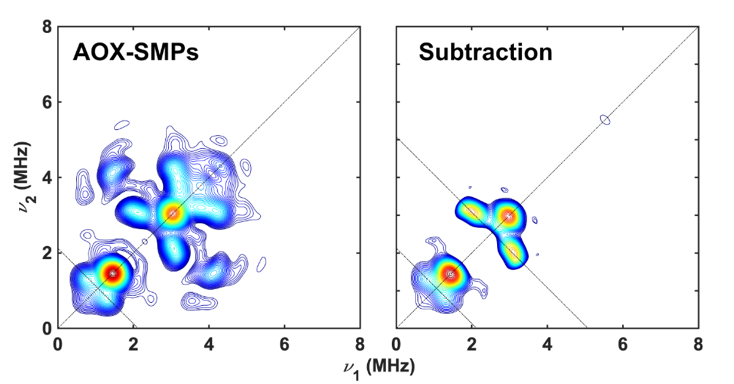
**

**Figure S5: HYSCORE spectrum and subtraction of carboxin inhibited AOX-SMPs.** Measurements and subtractions were performed as in Figure 7, with the subtraction spectrum showing the difference spectrum between the left-hand-side of Figure S5 and Figure 7B. Samples contained 100 µM carboxin. The AOX sample shown here does not contain antimycin A, so the subtraction represents an antimycin A sensitive ^14^N modulation.
