# Supplementary material for: Using a chimeric respiratory chain and EPR spectroscopy to determine the origin of semiquinone species previously assigned to mitochondrial complex I
Source: BMC Biol. 2020 May 20;18:54. doi: 10.1186/s12915-020-00768-6 (PMC7238650; doi:10.1186/s12915-020-00768-6)
Supplement: Supplementary file 6 — Summary of the parameters used to simulate 14N HYSCORE spectra. Table S1. Hyperfine and quadrupole parameters for 14N interactions in SMPs and AOX-SMPs. [file 12915_2020_768_MOESM6_ESM.docx]

1. **Summary of the parameters used to simulate ^14^N HYSCORE spectra**

The hyperfine and quadrupole parameters determined from this work are summarised in the table below, along with the values known for isolated complex III SQ_i_.

**Table S1: Hyperfine and quadrupole parameters for ^14^N interactions in SMPs and AOX-SMPs.** See Methods for a definition of the parameters.

|  | **Hyperfine** | | **Quadrupole** | |
| --- | --- | --- | --- | --- |
|  | ***A*_iso_ (MHz)** | ***T* (MHz)** | ***κ* (MHz)** | ***η*** |
| **SMPs (Antimycin A sensitive)** | 0.54 | -0.2 | 0.42 | 0.18 |
| ***Rhodobacter* Complex III Q_i_ site [44]** | 0.7 | -0.14 | 0.36 | 0.17 |
| **AOX-SMPs (Antimycin A insensitive)** | 1.0 | -0.08 | 0.8 | 0.6 |
