# Supplementary material for: Using a chimeric respiratory chain and EPR spectroscopy to determine the origin of semiquinone species previously assigned to mitochondrial complex I
Source: BMC Biol. 2020 May 20;18:54. doi: 10.1186/s12915-020-00768-6 (PMC7238650; doi:10.1186/s12915-020-00768-6)
Supplement: Supplementary file 7 — HYSCORE spectra of AOX-SMPs at different field positions. Figure S6. Echo-detected field sweep and HYSCORE spectroscopy of oxygen supplemented carboxin- and antimycin A-treated AOX-SMPs. [file 12915_2020_768_MOESM7_ESM.docx]

1. **HYSCORE spectra of AOX-SMPs at different field positions**

To ascertain that the ‘backbone ^14^N’ couplings observed do not originate from Fe-S cluster N1b, measurements were conducted off resonance from the SQ EPR signal:

**
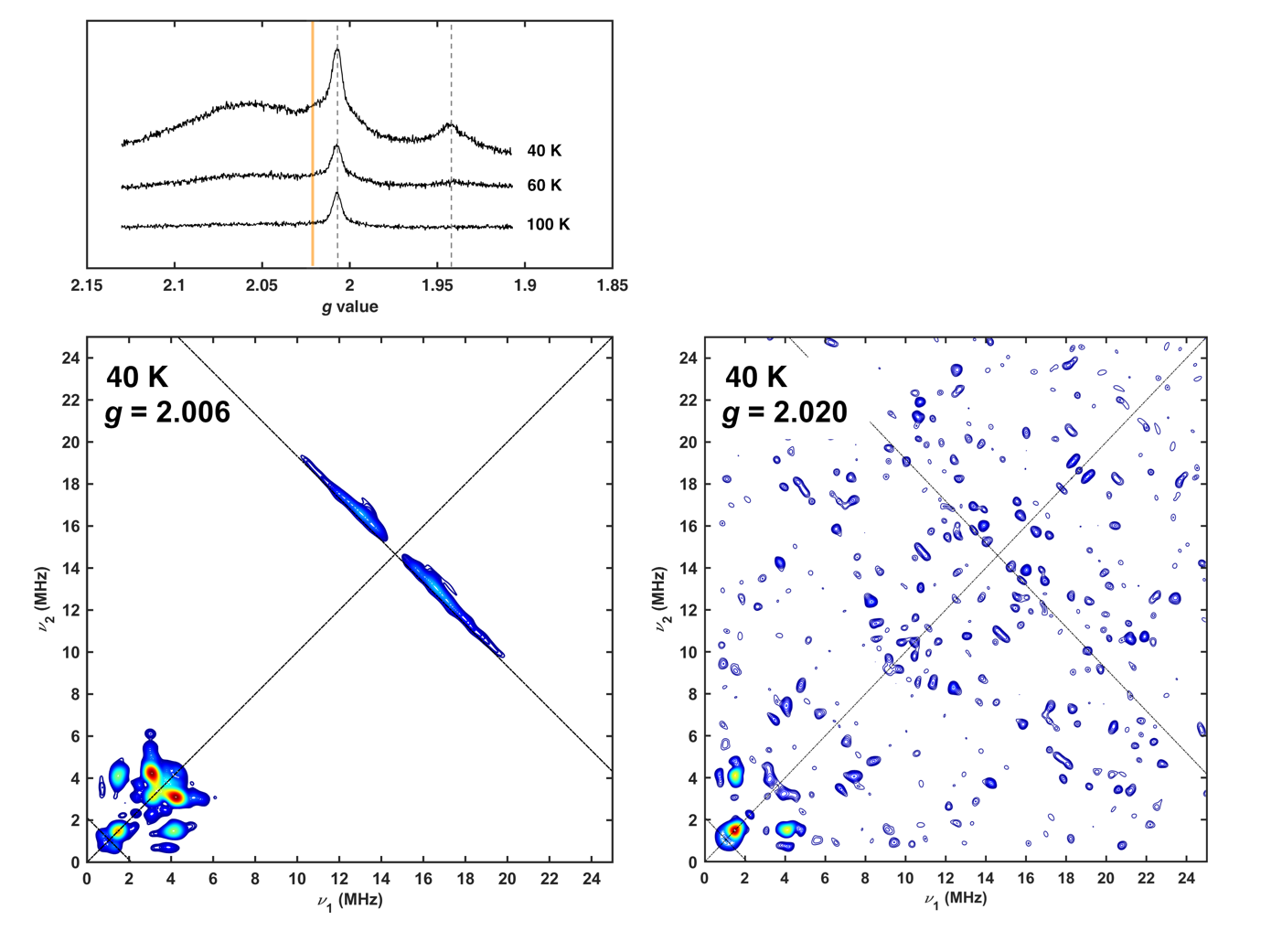
**

**Figure S6: Echo-detected field sweep and HYSCORE spectroscopy of oxygen supplemented carboxin- and antimycin A-treated AOX-SMPs.** (Top) Temperature dependence of echo-detected field sweeps of AOX-SMPs. Measurements were performed with a two-pulse sequence (π/2–τ–π–echo) with π/2 = 16 ns, π = 32 ns, τ = 200 ns. Shot repetition times and shots per point were kept constant across the temperature range at 2 ms and 50, respectively. Vertical dashed lines indicate the maximum intensity of the semiquinone signal (*g* = 2.006) and the N1b signal (*g* = 1.94). The orange line indicates the field position of the off SQ resonance HYSCORE measurement. (Bottom left) 40 K HYSCORE (+,+) quadrant of the *g* = 2.006 signal of AOX-SMPs. (Bottom right) 40 K HYSCORE (+,+) quadrant of the off SQ resonance *g* = 2.006 signal of AOX-SMPs. Differences in S/N are due to the decrease in spin concentration at *g* = 2.020 and the lower number of accumulated scans. HYSCORE measurement conditions as in Figure 7.
