# Supplementary material for: Using a chimeric respiratory chain and EPR spectroscopy to determine the origin of semiquinone species previously assigned to mitochondrial complex I
Source: BMC Biol. 2020 May 20;18:54. doi: 10.1186/s12915-020-00768-6 (PMC7238650; doi:10.1186/s12915-020-00768-6)
Supplement: Supplementary file 8 — Complete HYSCORE spectrum of the g ~ 2 signal in complex II inhibited SMPs. Figure S7. Echo-detected field sweep and HYSCORE spectroscopy of oxygen supplemented SMPs treated with carboxin. [file 12915_2020_768_MOESM8_ESM.docx]

1. **Complete HYSCORE spectrum of the *g* ~ 2 signal in complex II inhibited SMPs**

The absence of any significant amounts of N1b in the complex II inhibited SMP sample lends further evidence to the ‘backbone ^14^N’ couplings originating from the *g* ~ 2 SQ species.


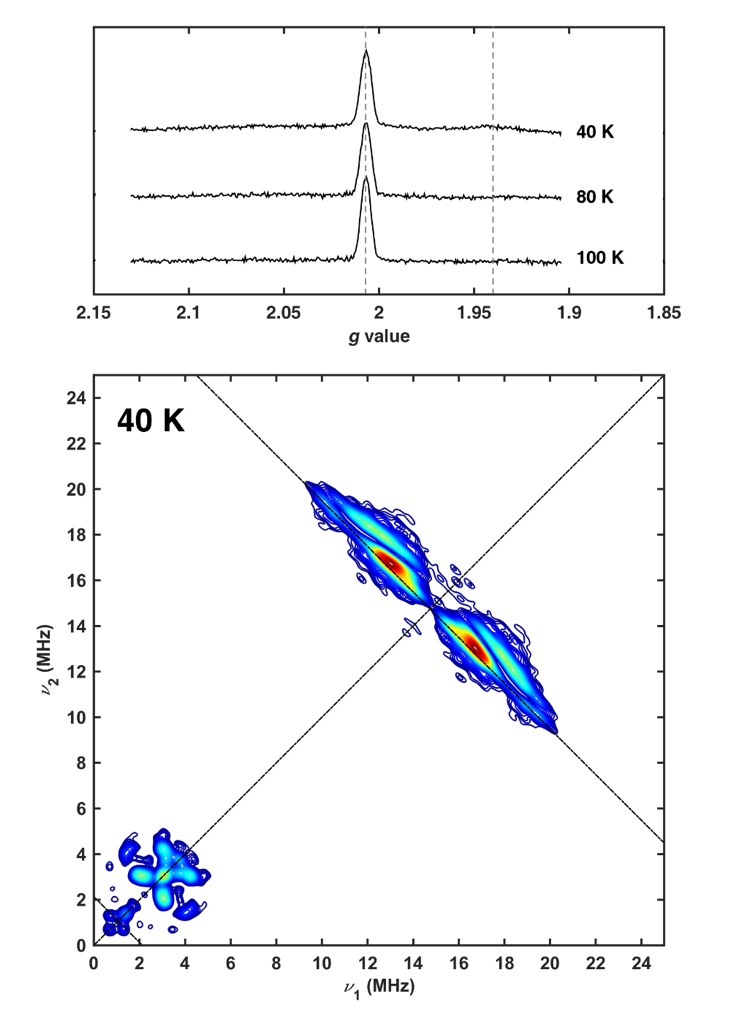


**Figure S7: Echo-detected field sweep and HYSCORE spectroscopy of oxygen supplemented SMPs treated with carboxin.** (Top) Temperature dependence of echo-detected field sweeps of SMPs. Measurements performed with a two-pulse sequence (π/2–τ–π–echo) with π/2 = 16 ns, π = 32 ns, τ = 200 ns. Shot repetition times and shots per point were kept constant across the temperature range at 2 ms and 50, respectively. Vertical lines indicate the maximum intensity of the semiquinone signal (*g* = 2.00) and the N1b signal (*g* = 1.94) (Bottom) 40 K (+,+) quadrant of the *g* = 2.006 signal of carboxin inhibited SMPs with oxygen supplementation. HYSCORE measurement conditions as in Figure 7.
