# Supplementary material for: Using a chimeric respiratory chain and EPR spectroscopy to determine the origin of semiquinone species previously assigned to mitochondrial complex I
Source: BMC Biol. 2020 May 20;18:54. doi: 10.1186/s12915-020-00768-6 (PMC7238650; doi:10.1186/s12915-020-00768-6)
Supplement: Supplementary file 9 — Investigating the ‘split N2’ signal. Figure S8. The effect of uncouplers on the semiquinone EPR signal in uninhibited SMPs undergoing NADH:O2 turnover. [file 12915_2020_768_MOESM9_ESM.docx]

1. **Investigating the ‘split N2’ signal**

As summarised in Figure S9 below, we could not find any evidence for the presence of a ‘split N2’ *g*_z_ EPR signal in samples that contained large *g* ~ 2 signals.

**
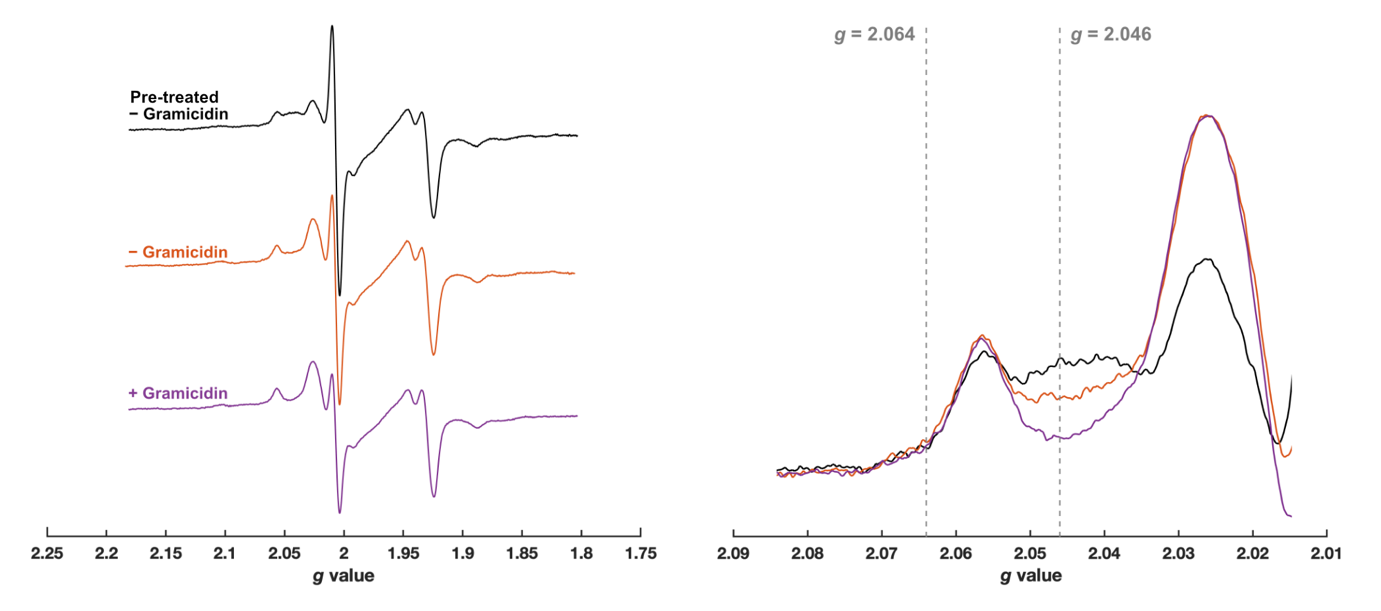
**

**Figure S8: The effect of uncouplers on the semiquinone EPR signal in uninhibited SMPs undergoing NADH:O_2_ turnover.** Samples (25 mg/mL) were reduced by the addition of 15 mM NADH under continuous oxygen flow. The NADH pre-treated sample (black) was mixed with 2 mM NADH and incubated aerobically for 2 minutes prior to sample preparation. The uncoupled sample (purple) was incubated with 40 µM gramicidin for 30 minutes before sample preparation. Both the coupled (orange) and uncoupled (purple) samples contained an equivalent amount of DMSO (0.4%). Right panel shows the *g*_z_ region of the N2 line (with dotted lines to indicate the *g* values of the split N2 signal reported in the literature [8]). The feature observed at *g =* 2.046 is likely to arise from reduction of complex II, concomitant with the decrease of the S3 cluster signal (*g* = 2.022). No obvious feature was observed at *g* = 2.064.
